# Scenario analysis of ecosystem service changes and interactions in a mountain-oasis-desert system: a case study in Altay Prefecture, China

**DOI:** 10.1038/s41598-018-31043-y

**Published:** 2018-08-28

**Authors:** Qi Fu, Ying Hou, Bo Wang, Xu Bi, Bo Li, Xinshi Zhang

**Affiliations:** 1 College of Resources Science & Technology, Faculty of Geographical Science, Beijing Normal University, Beijing, 100875 China; 2 Key Laboratory of Watershed Geographic Sciences, Nanjing Institute of Geography and Limnology, Chinese Academy of Sciences, Nanjing, 210008 China; 3 State Key Laboratory of Urban and Regional Ecology, Research Center for Eco-Environmental Sciences, Chinese Academy of Sciences, Beijing, 100085 China; 4 Institute of Ecology and Rural Environment Planning, Chinese Academy for Environmental Planning, Beijing, 100012 China; 5 Institute of Botany, Chinese Academy of Sciences, Beijing, 100093 China

## Abstract

Scenario analysis of ecosystem services (ES) can provide a scientific basis for ecosystem management. The objective of this study was to reveal the effects of future land use scenarios on ES in a mountain-oasis-desert system (MODS). We first simulated land use changes for the period of 2015–2035 in Altay Prefecture under three different scenarios: business as usual (BAU), economic development (ED), and ecological conservation (EC). We then evaluated water yield (WY), crop production (CP), soil conservation (SC), sand fixation (SF), carbon sequestration (CS), and aesthetic value (AV) and investigated the multiple interactions among ES at the regional and grid scales. The results showed that SC, CS, and AV continually increased, WY continually decreased under the three scenarios. Our study revealed that the multiple interactions among ES were spatially heterogeneous in the MODS and the spatial heterogeneities changed across scenarios. The locations of and causes for the formation of the multiple interactions among ES were identified based on spatial analysis. This information can help decision-makers develop targeted and differentiated ecosystem management strategies. This study can increase the understanding of the multiple interactions among ES. Our findings can provide a reference for studies of other regions with the MODS structure.

## Introduction

Ecosystem services (ES) are the basis of human survival and social development and are closely related to human well-being^1^. Since ES were first proposed, researchers have conducted many studies on the definition, classification, and evaluation of ES^2,3^. Many research achievements have greatly promoted the public’s understanding of ES, and its concepts and methods have been gradually applied to the formulation of policy for ecosystem management^4,5^. However, as in-depth ES research continues, a mere assessment of its value is insufficient to meet the demand for decision-making^6^. The trade-offs, modeling and scenario planning, bundling, and scaling of ES are gradually becoming hot topics of current research^7^.

With global population growth and rapid social development, the human demand for ES has been continuously increasing^8^. When stimulated by interests, people often focus only on the production function of ecosystems and ignore their ecological functions. Many case studies have shown that agricultural extension, urbanization, and grazing have caused carbon storage loss, decreased water quality, and reduced biodiversity^9–11^. With the intensification of human activities, 60% of the global ES has been degraded^12^. Therefore, the adoption of effective ecosystem management strategies is critical to reduce the adverse effects of human activities.

To achieve rational ecological management, understanding the interactions among ES is important^13^. The interactions among ES are usually reflected as trade-offs and synergies^14^. Trade-offs occur when the provision of one or some ES increases at the cost of other services; synergies arise when multiple ES increase simultaneously^14–16^. Many studies have reported trade-offs between provisioning and regulating services, or between provisioning and cultural services. Whereas the interactions between different regulating services and between different cultural services often display synergies^17–19^. Among the trade-offs, those between some regulating services and food production have drawn the most attention^20^. For example, noticeable trade-offs have been found between food production and water quality regulation in the Great Barrier Reef, Australia^21^. Another example in Beijing, China has found that food provision has negative correlations with carbon storage, water purification and habitat provision^22^. Furthermore, erosion control is significantly negatively correlated with the cultivated crops in southeast Spain^23^. The interactions among ES can differ between different regions because of landscape heterogeneity across the regions and the distinct ecosystem management strategies of the regions (e.g. a trade-off between food production and carbon stocks are identified in the Sanjiang Plain of China^24^, while a synergy between these two services is found in the Loess Plateau of China^25^). In addition, the uncertainties in ES assessment may also lead to different results in the study of ES interactions^26^.

The methods for analyzing the ES interactions can be broadly classified into four categories: spatial overlay analysis^27,28^, correlation analysis^29,30^, ES-bundle analysis^19,31,32^, and scenario analysis^33–38^. Among them, scenario analysis is currently one of the most common methods used in ES trade-off and synergy studies, which can usually be used in combination with other methods^16^. By setting up alternative land use scenarios and calculating the changes in and interactions among ES, this method can help improve land use policy-making^36,39^. Moreover, scenario analysis is used to explore the impact of policy or climate change on ES^35^. However, there are still some limitations in previous studies. First, many studies are too subjective in the setup of the scenarios^36–38^, often leading to conclusions that are not sufficiently convincing. Second, the ecosystem has diverse functions and thus provides multileveled services to humans; the intertwining of various services has become a challenge in ecosystem management^40^. However, most of the existing studies consider only the pairwise interactions among ES^29,39,41,42^ and, therefore, lack investigation into multiple interactions among ES^43^, thereby failing to meet the needs of scientific decision-making. Third, to improve ecosystem management, decision-makers and scientists need to know the specific location where ES trade-offs and synergies occur. However, few studies have identified the locations where multiple interactions among ES occur^44^.

**Figure 1:**
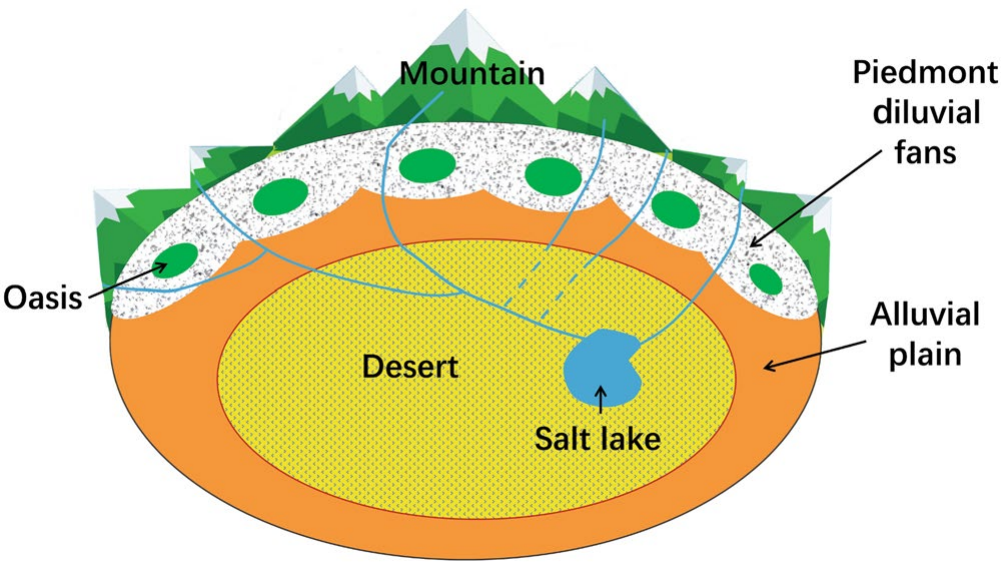
Schematic diagram of the MODS landscape.

In terms of study region, current ES research focuses more on cities, urban-rural areas, and watersheds while not paying enough attention to arid regions. The uneven distribution of mountains, oases, and deserts is a basic feature of the natural geography of the arid region of Central Asia. This combination of terrestrial ecosystems is defined as the mountain-oasis-desert system (MODS)^45^ (Fig. 1). The MODS is not only a regional geological and landform framework but also largely determines the climatic conditions, ecosystem patterns, and human activities in the region. In addition, the mountains, oases and deserts are closely connected by energy material, and value flows^45^. The formation of oases and deserts is inseparable from the supply of water and sediment by mountains. In current ES-related research, many studies focus on the mountains^46,47^, oases^48,49^, or deserts^50,51^ separately, but few examine the complex system of the MODS.

To narrow these gaps in research, we used the MODS in Altay Prefecture, China, as an example, focused on the multiple interactions among ES in the area, and conducted a scenario analysis of the ES changes and interactions. To conduct a more realistic simulation for the land use change scenarios, we applied Markov chain and FLUS (future land use simulation) composite models^52^. In addition, our study spatially identified the multiple interactions among ES in Altay Prefecture. The main objectives of this study are to (1) explore the ES changes and interactions under different land use change scenarios in regions with the MODS structure and (2) analyze the causes for the changes in ES and for the formation of the multiple interactions among ES. This work can increase the understanding of the multiple interactions among ES. In addition, our findings can provide a reference for studies of other regions with the MODS structure.

## Results

### Markov chain and FLUS models validation

By comparing the simulated values with the actual values of land use types in 2015, we found that the built-up area had the largest error (−1.02%), whereas the errors for the other land use types were all less than 1% (see Supplementary Table S4). This result shows that using the Markov chain model to simulate the land use change in Altay Prefecture achieves the required precision. The ROC values of various land use types—cropland, forest, grassland, water, built-up area, and bare land—were 0.935, 0.956, 0.928, 0.848, 0.832, and 0.96, respectively (see Supplementary Fig. S2). All ROC values were greater than 0.8, which means that the driving factors sufficiently explained the land use allocation. The kappa coefficient was 0.94, indicating that the use of the FLUS model to simulate the land use change in Altay Prefecture is reasonable (see Supplementary Table S5). The simulated and actual land use patterns for 2015 can be found in Supplementary Fig. S3.

### Land use change

**Figure 2:**
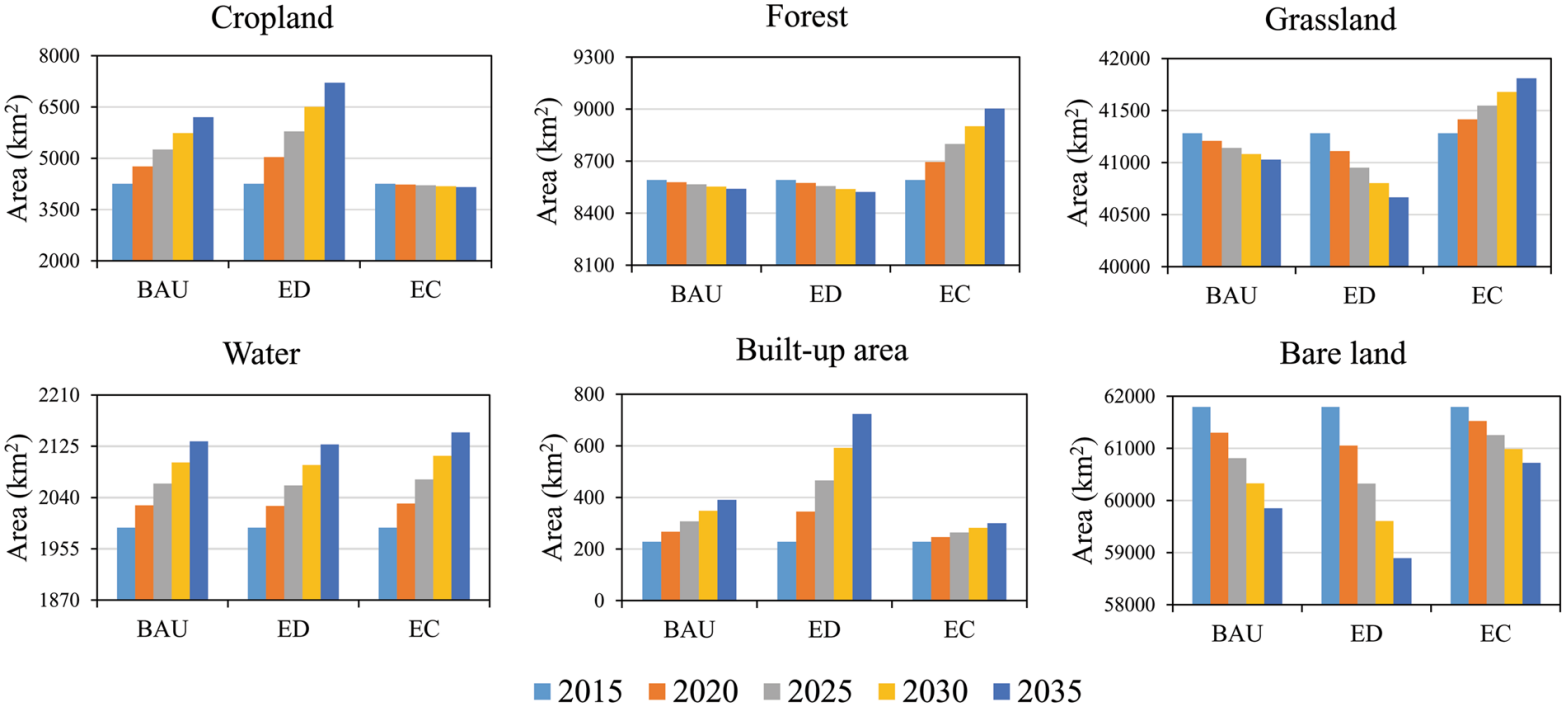
Quantitative changes in each land use type from 2015 to 2035 under the different scenarios.

**Figure 3:**
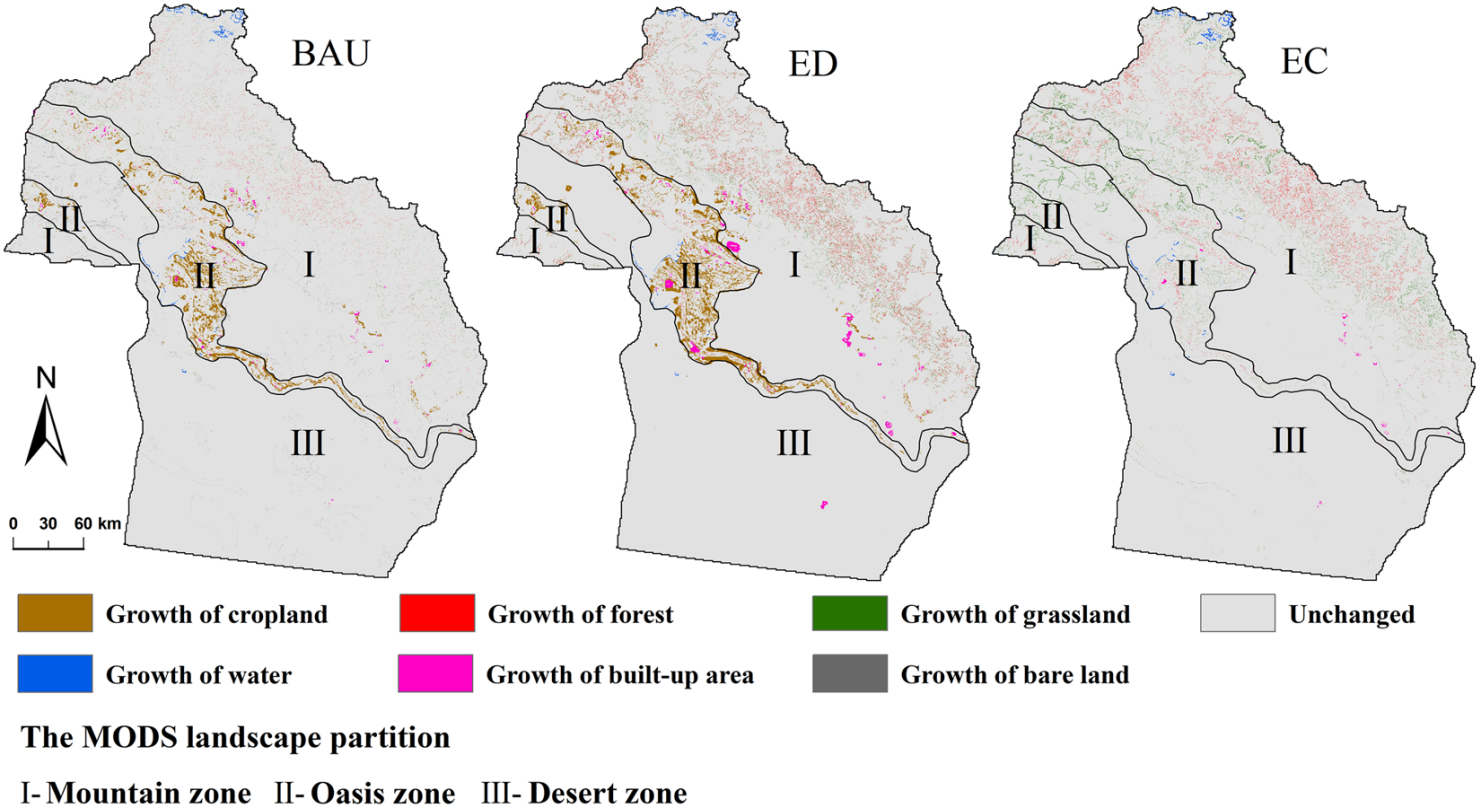
Changes in the spatial pattern of land use from 2015 to 2035 under the different scenarios. Maps were generated using ArcGIS 10.2 for Desktop (http://www.esri.com/software/arcgis).

During the period from 2015 to 2035, the cropland area in Altay Prefecture increased substantially under the BAU and ED scenarios (1963 km^2^ and 2959 km^2^, respectively) and remained virtually unchanged under the EC scenario (Fig. 2). The area of forest and grassland increased by 410 km^2^ and 526 km^2^, respectively, under the EC scenario, and showed a decreasing trend under both the BAU and ED scenarios. Water showed an increasing trend in all three scenarios. The built-up area increased by 495 km^2^ under the ED scenario and increased by 166 km^2^ and 72 km^2^ under the BAU and EC scenarios, respectively. Bare land area showed a substantial decrease under all three scenarios, with the largest decrease under the ED scenario and the smallest decrease under the EC scenario. From the perspective of spatial distribution, under the BAU and ED scenarios, cropland in the oasis zone increased greatly, and forest, grassland, water, and built-up area in the mountain zone increased moderately (Fig. 3). The increase in built-up area was more obvious under the ED scenario. Under the EC scenario, forest in the mountain and oasis zones and grassland in the desert zone increased significantly due to conservation measures.

### ES changes

**Figure 4:**
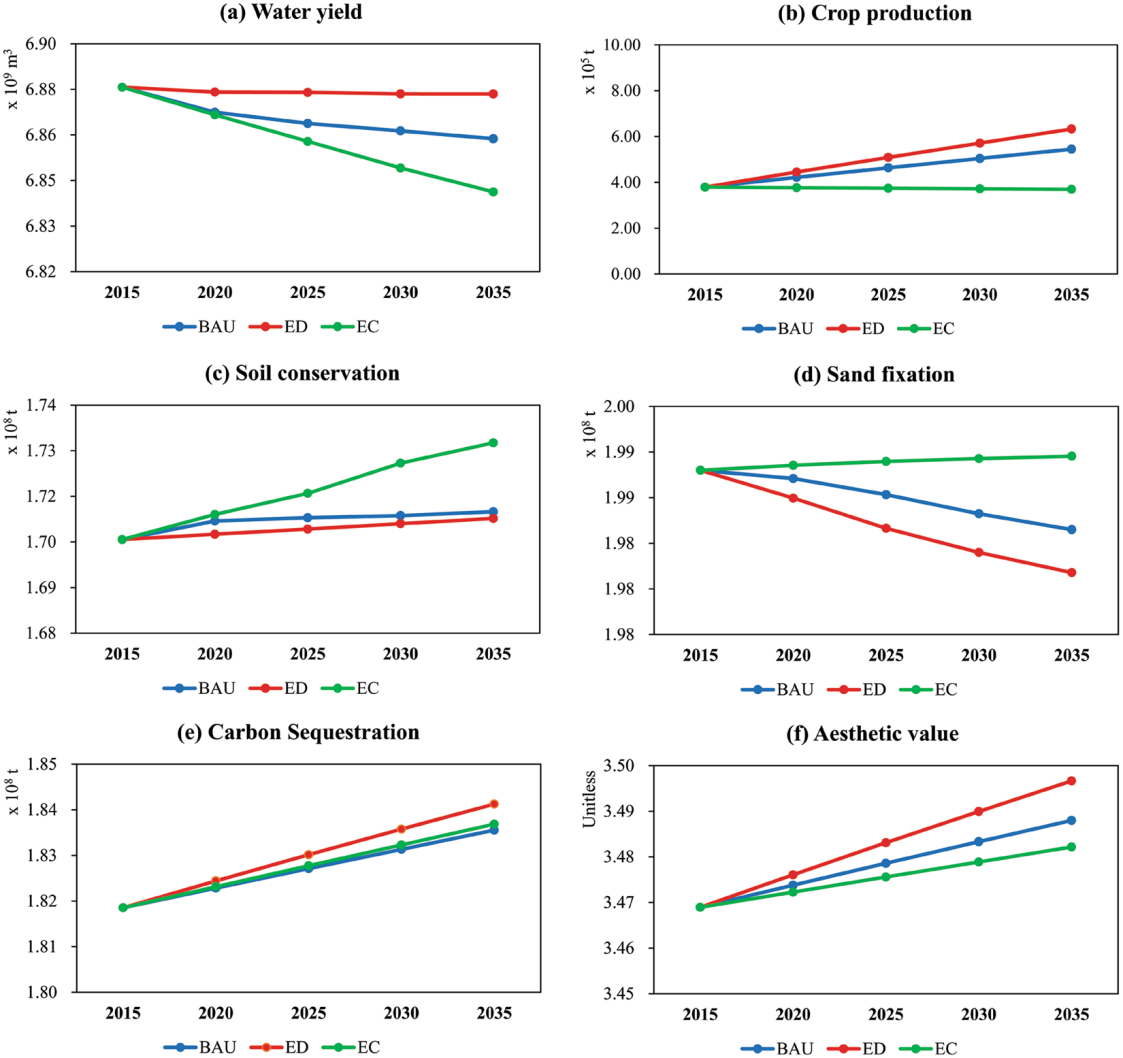
Changes in the total amount of ES from 2015 to 2035 under the different scenarios.

**Figure 5:**
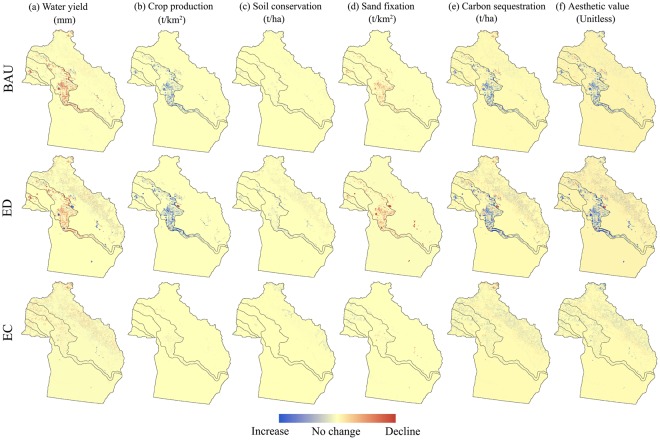
Spatial distributions of the changes in ES from 2015 to 2035 under the different scenarios. Maps were generated using ArcGIS 10.2 for Desktop (http://www.esri.com/software/arcgis).

From 2015 to 2035 in Altay Prefecture, the WY declined under all three scenarios, among which the EC scenario showed the greatest reduction in WY (Fig. 4a). The reduction in WY under the BAU and ED scenarios was observed mainly in the oasis zone, while under the EC scenario, the reduction in WY was scattered and widely distributed throughout the mountain, oasis, and desert zones (Fig. 5a). CP increased most under the ED scenario, followed by the BAU scenario, with a large increase in cropland in all scenarios (Fig. 4b). The areas where CP increased were distributed mainly in the oasis zone and its surrounding mountain and desert zones (Fig. 5b). SC increased under all three scenarios (Fig. 4c). The increase in SC was distributed mainly in the oasis zone under the BAU and ED scenarios, while under the EC scenario, the increase in SC was found mainly in the mountain zone at high altitude (Fig. 5c). SF first showed an increasing trend under the EC scenario, and a continually decreasing trend under the BAU and ED scenarios (Fig. 4d). The decrease in SF occurred mainly in the oasis zone (Fig. 5d). CS increased continuously under all three scenarios (Fig. 4e). Although the total quantity of CS in the entire study area increased, this service decreased in some areas of the mountain and oasis zones (Fig. 5e). The total value of AV also increased continuously under all three scenarios, with the largest increase occurring under the ED scenario (Fig. 4f). The increase in AV was distributed mainly in the mountain and oasis zones (Fig. 5f).

### ES interactions

**Figure 6:**
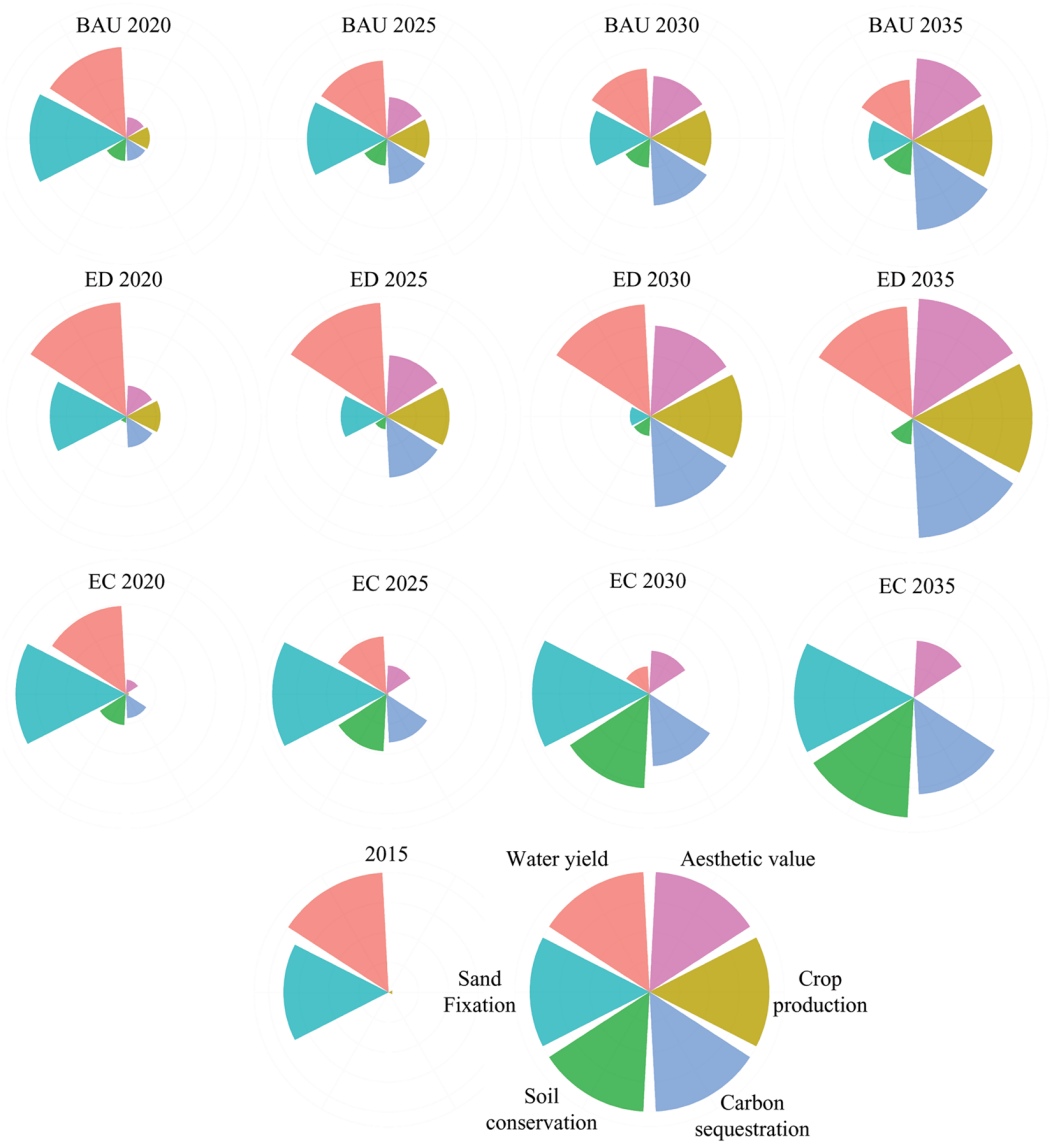
Interactions among ES at regional scale under the different scenarios during 2015–2035. These figures were generated using R studio software version 1.1.383 (https://www.rstudio.com/).

Figure 6 depicts the multiple interactions among ES from the perspective of the study area as a whole unit. Under the BAU scenario, WY and SF showed simultaneous continuous decreases, and CP, SC, CS and AV showed a simultaneous continuous increases. This phenomenon means that the CP, SC, CS and AV services had synergistic interactions. Meanwhile, the two services WY and SF both had trade-off interactions with the four services AV, CP, CS and SC. Under the ED scenario, SF gradually decreased; AV, CP, CS, and SC showed simultaneous continuous increases; and WY remained virtually unchanged from 2015 to 2035. This result indicates that synergistic interactions occurred among AV, CP, CS, and SC throughout the years. Furthermore, these four services showed trade-off interactions with SF. Under the EC scenario, WY continuously decreased; SF, AV, SC, and CS simultaneously underwent a continuous increase; and CP remained virtually unchanged from 2015 to 2035. Synergies occurred among SF, AV, SC, and CS, and these four services exhibited trade-off interactions with WY.

**Figure 7:**
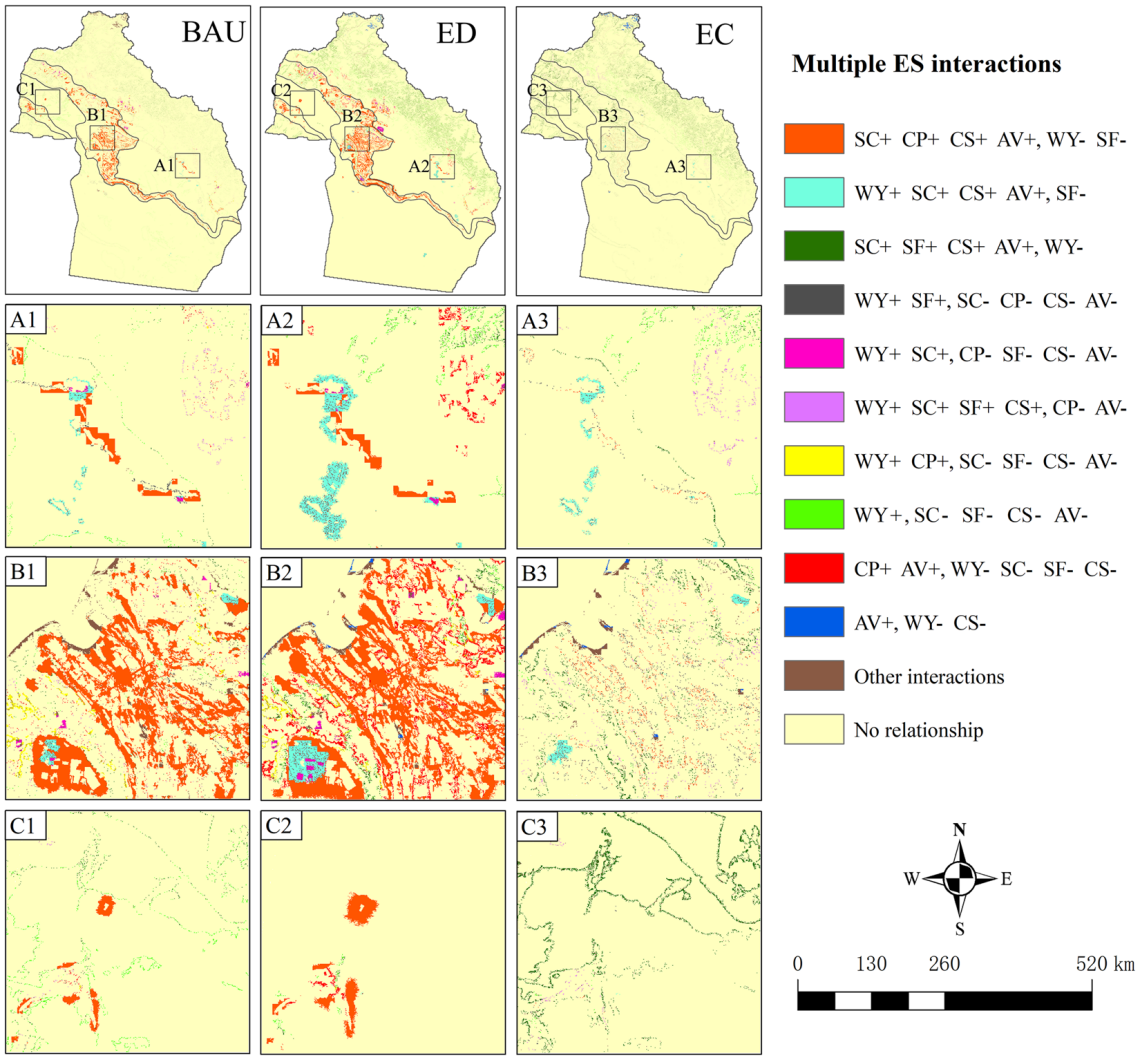
The spatial patterns of multiple interactions among ES under the different scenarios during 2015–2035. Maps were generated using ArcGIS 10.2 for Desktop (http://www.esri.com/software/arcgis). Because the area of Altay Prefecture is approximately 118,000 km^2^, only those multiple interactions occurring in pixels (100 m × 100 m) with a summed area of over 40 km^2^ are shown in this figure, and the others are collectively named “other interactions”; ‘+’ indicates an increase of the ES; ‘−’ indicates a reduction. For example, “SC+ CP+ CS+ AV+, WY− SF−” indicates that SC, CP, CS, and AV increased simultaneously (synergies), WY and SF decreased simultaneously, and the two services WY and SF both exhibited trade-off interactions with the four services SC, CP, CS, and AV.

The interactions among ES were different on the grid scale across Altay Prefecture (Fig. 7). Under the BAU and ED scenarios, the trade-offs among the two services WY and SF (simultaneous decreases) and the four services SC, CP, CS, and AV (simultaneous increases, suggesting synergies) occurred in many areas of the mountain and oasis zones. Under the ED scenario, the area in which these interactions occurred was larger compared to the BAU scenario, and more trade-offs between the four services WY, SC, CS, and AV (simultaneous increases, meaning synergies) and SF occurred in the mountain zone; however, few multiple interactions among ES occurred in the desert zone. Under the BAU scenario, trade-offs between WY and the four services SF, SC, CS, and AV occurred in the desert zone. Under the EC scenario, synergies among SC, SF, CS, and AV (simultaneous increases) and trade-offs between these four services and WY occurred widely in the mountain, oasis, and desert zones. In addition, trade-offs among the four services WY, SC, SF, and CS (simultaneous increases, meaning synergies) and the two services CP and AV (simultaneous decreases) occurred in the mountain-oasis ecotone.

## Discussion

### ES changes

Our study found that except for SC and SF, the values of all services in 2035 were the highest under the ED scenario (Fig. 4). This finding is inconsistent with those of some existing studies, which showed that most ES would increase greatly under conservation scenarios^34,36,37^. This result is mainly due to the distinct MODS geographical pattern and human activities in arid regions. In Altay Prefecture, mountains and deserts account for a large area of land, and oases are located only on the two sides of the narrow river in the middle of the study area^53^. Due to their flat terrain, deep soil layers, and good irrigation conditions, the oases and their surrounding areas are often reclaimed as cropland. Under the ED scenario, a large increase in cropland directly led to a substantial increase in CP. The increase in built-up area led to an increase in impervious surface, thereby resulting in an increase in WY in these areas (Fig. 5a) and a larger total amount of WY than that in the BAU and EC scenarios. In many studies, the increase in cropland was due to encroachment of forest or grassland^38,54,55^. In our study, however, the increase in cropland was mainly due to the conversion of bare land, since the oasis margin was desert. The carbon pool stores in cropland are much larger than those in bare land (see Supplementary Table S11); hence, a large amount of bare land was reclaimed as cropland, resulting in the largest amount of CS under the ED scenario. Based on the results of an aesthetic value survey, we found that the local residents (most of them are Kazakh) prefer cropland to grassland. Although the score of alpine meadow was high, the score of desert grassland was low, and its area was much larger than that of alpine meadow, resulting in a great decrease in the average score of the whole grassland category after the area weighting calculation (see Supplementary Section 2.6). The low score of desert grassland may be because of that most local residents are engaged in animal husbandry and therefore do not like desert grasslands with low productivity. Moreover, in Altay Prefecture, most of the crops in the cultivated land are of wheat, corn and sunflowers, which are neater and more pleasing to the eye and thus preferred by local people. This explains why the increase in AV was lower than that under the BAU and ED scenarios, even though the area of grassland increased significantly under the EC scenario.

### ES interactions

For the entire study area, we found that the multiple interactions among ES under the different scenarios have different characteristics (Fig. 6). For example, under the ED scenario, SF decreased continually and exhibited trade-off interactions with the four services SC, CP, CS, and AV. This phenomenon indicates that the maintenance of the SF service should be particularly emphasized when implementing economic development policies in Altay Prefecture. Under the EC scenario, the WY decreased continually and exhibited trade-off interactions with the four services SC, SF, CS, and AV, indicating that vegetation recovery may lead to water resource shortage in the region. Flower diagram analysis helps to formulate policies at the national or regional level, but it cannot accurately explain to managers the location of and causes for the formation of multiple interactions among ES^32,56^. The study by Haase *et al*. showed that for planning purposes, only analysis at the grid scale is meaningful^57^. In this study, we identified the locations where multiple interactions among ES occurred (Fig. 7) and further analyzed the causes for the formation of these interactions (see Supplementary Table S13). Conducting studies on the spatial heterogeneity of the multiple interactions among ES could help to provide a targeted scientific basis for decision-makers to manage different regions.

Our analyses revealed that under the BAU and ED scenarios, the conversion of bare land to cropland leads to trade-offs between the two services WY and SF and the four services SC, CP, CS, and AV in many areas of the mountain and oasis zones. In Altay Prefecture, agriculture is an important source of income for residents, second only to animal husbandry. In general, the larger the cropland area, the more income people can earn. The desert of the MODS in Altay Prefecture is mainly formed by the accumulation of sediments and gravels^45^. There are large areas of loamy deserts in the oasis zone and its surrounding areas, which are often reclaimed as cropland by local residents. However, the reclamation of cropland from bare land will lead to more actual evapotranspiration and less WY. At the same time, cropland reclamation will destroy the soil crust and reduce the SF service^58^. Without the SF service provided by the surrounding ecosystems, the cropland would eventually be overtaken by deserts. In addition, we found that the cropland reclamation leads to the increase of SC and a synergistic relationship between SC and CP, which is inconsistent with other studies^59–61^. In these studies, the increased cropland is usually converted from forest, grassland or wetland, which often leads to increased soil erosion. In our study, the conversion of bare land to cropland enhanced vegetation cover and soil conservation measures, resulting in the increase of SC service. The conversion of cropland or bare land to built-up area will also result in a reduction in SF; therefore, such land se activities should be prohibited in the oasis margin. Under the EC scenario, a large area of grassland was converted to forest in the mountain and oasis zones. These conversions led to trade-offs between WY and four services SC, SF, CS, and AV. Precipitation in the mountain zone is abundant, especially the heavy rainfall that occurs in summer, which can easily lead to geological disasters such as debris flow and landslides. The increase in forests in the mountain zone can not only enhance SC but also reduce surface runoff to a certain extent, thereby reducing the probability of debris flow and landslides. Moreover, the forests have strong carbon sequestration capacity and can provide high landscape aesthetic value. Therefore, afforestation in the mountain zone is a preferable approach of ecological conservation. However, extensive afforestation in the oasis zone is irrational in the study area. In the oasis zone, there is usually low precipitation, and the groundwater level is near the soil surface. The strong transpiration of trees would cause the groundwater level to rise; with the evaporation of water, the salt in the groundwater would remain on the surface, resulting in soil salinization^62^. Therefore, large-scale afforestation not only would cause a waste of water resources but also might cause soil salinization due to strong transpiration. Our study showed that the conversion of bare land to grassland improved the SC, SF, CS, and AV services in the cost of WY in the desert zone. The desert zone is the source of wind erosion, where the growing of grass would greatly increase SF. In contrast, the grass types that can grow in desert zone have considerably low transpiration capacity^63^, which would only slightly reduce WY. Therefore, we consider it a reasonable approach of ecological conservation to convert bare land to grassland in the desert zone^42^.

## Conclusion

This study simulated land use change scenarios and explored the multiple interactions (i.e., trade-offs and synergies) among ES in the MODS of Altay Prefecture. Moreover, we revealed the causes for the formation of the interactions. The results showed that SC, CS, and AV continually increased, WY continually decreased under the three scenarios. The changes of CP and SF showed different trends under different scenarios. The multiple interactions among ES exhibit different patterns for the entire Altay Prefecture under different scenarios. In addition, the interactions were spatially heterogeneous on the grid scale across Altay Prefecture. Under the BAU and ED scenarios, the conversion of bare land to cropland in the mountain and oasis zones led to a continuous decrease in WY and SF and caused them to exhibit trade-off interactions with the four services SC, CP, CS, and AV. Under the EC scenario, the conversion of grassland to forest in the mountain and oasis zones and the conversion of bare land to grassland in the desert zone caused SC, SF, CS, and AV to continuously increase and to exhibit trade-off interactions with WY. This information can help decision-makers develop targeted and differentiated ecosystem management strategies. This study can increase the understanding of the multiple interactions among ES. In addition, our findings could provide a reference for ecosystem management in other areas with the MODS structure.

## Methods

### Study Area

**Figure 8:**
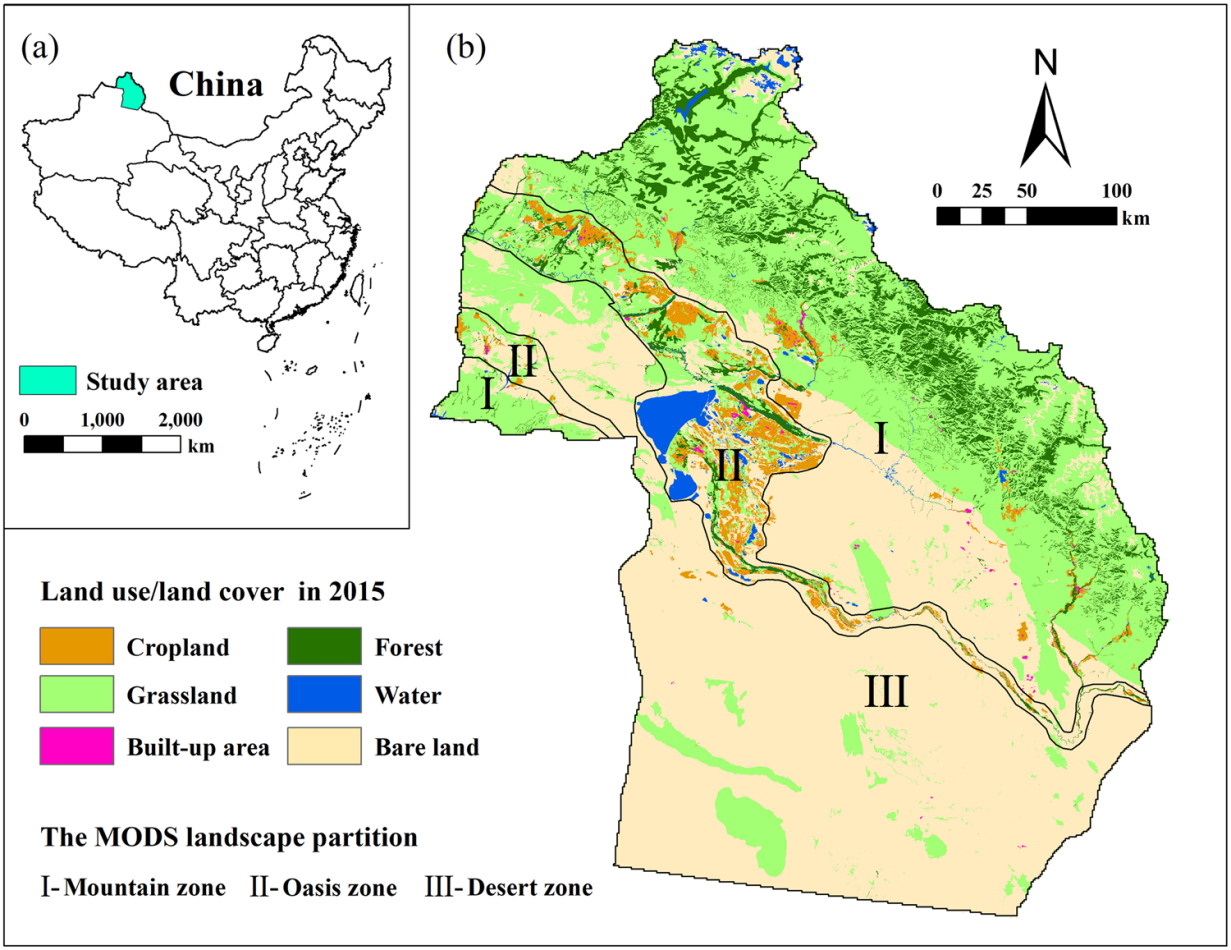
(**a**) The location of Altay Prefecture in China; (**b**) the land use/land cover (LULC) pattern and the geographical division of Altay Prefecture. Maps were generated using ArcGIS 10.2 for Desktop (http://www.esri.com/software/arcgis).

Altay Prefecture is located in the extreme north of Xinjiang, China, between 85°31′57″E and 91°01′15″E and between 44°59′35″N and 49°10′45″N. It is bordered by Kazakhstan, Russia, and Mongolia. The total area of the prefecture is approximately 118,000 km^2^, and the total population is approximately 675,900 (2015). The terrain descends from north to south and has an obvious steppe-like topography. Altay Prefecture has a typical temperate continental climate. The average annual temperature ranges from 0.7–4.9 °C and decreases with an increase in altitude and latitude; the average annual precipitation in the study area is approximately 139.3–268.4 mm and decreases with a decrease in altitude. The soil distribution in the study area has an obvious vertical band spectrum, and most of the soil is characterized by a thin soil layer, a coarse texture, and the ability to readily undergo salinization. These unique terrain, climate, and soil characteristics render Altay Prefecture a typical large-scale, multilevel, and regularly patterned “MODS”. As of 2015, the landscape of Altay Prefecture consists of 34.9% grassland, 7.3% forest, 3.6% cropland, 52.3% bare land, and 1.9% other land use/cover types (Fig. 8).

### Land use change simulation

Many models for simulating land use changes exist, but no single model can cover all the processes of land use changes^64–66^. In our study, Markov chain and FLUS models were used jointly to perform simulation of the land use changes (see Supplementary Fig. S1). The land demands under the different scenarios were obtained based on the Markov chain model. First, we analyzed the land use changes from 2000 to 2015, and obtained the initial transition probability matrix. Then, using the year 2015 as the initial year, we calculated the land demands under different scenarios by modifying the initial transition probability matrix^67,68^. The spatial allocation of land use was determined by using the FLUS model, which is created based on artificial neural network (ANN) and cellular automata (CA) models. The simulation accuracy of the FLUS model is higher than that of other well-accepted models (e.g., CLUE-S and CA models)^52^. The ANN model was used to calculate the suitability probability of each land use type; in this study, a total of 16 spatial driving factors were used for training the ANN model (see Supplementary Table S1). Combined with the suitability probability, conversion cost, and neighborhood weights, the CA model was used to simulate the spatial allocation of land use. The conversion cost and neighborhood weights were estimated based on historical land use data (see Supplementary Tables S2 and S3). The Markov chain model and FLUS model were run with Matlab software (www.mathworks.com) and GeoSOS-FLUS software (http://www.geosimulation.cn/FLUS.html), respectively.

In this study, three alternative potential land use change scenarios were developed, namely, business as usual (BAU), economic development (ED), and ecological conservation (EC), to detect the ES changes and interactions under these scenarios. The principles and goals of designing these scenarios were as follows:

(i) BAU scenario. This scenario assumed that the historical land use change trends are maintained; the land demand in the period of 2015–2035 can be calculated according to the initial transition probability matrix for the period of 2000–2015.
(ii) ED scenario. The ED scenario depicted a situation in which, to meet the demand of economic development, a large area of cropland is reclaimed, and the built-up area expands in this region. Referring to previous studies^67,69,70^, we calculated the land demands by modifying the conversion rate between certain land use types. In the period of 2015–2035, the conversion probabilities of forest, grassland, water and bare land into cropland increase by 50%, and the conversion probabilities of cropland, forest, grassland and bare land into built-up area increase by 200%.
(iii) EC scenario. This scenario described a situation in which the local government strengthens the protection of forest, grassland, and water; strictly controls the increase in cropland and built-up area; and encourages peasants to return cropland to forest, grassland, and lake. Similar to the ED scenario, we calculated the land demands for this scenario by modifying the conversion rate between certain land use types^67,69,70^. In the period of 2015–2035, the conversion probabilities of forest, grassland, and water into cropland and built-up area decrease by 100%; the conversion probabilities of bare land into cropland and built-up area decrease by 50%; and the conversion probabilities of cropland into forest, grassland, and water increase by 80%.

To test the effectiveness of the Markov chain model, we used the year 2000 as the initial year to simulate the land demand in 2015 and then compared it with the actual areas of each land use type in 2015. The FLUS model test was divided into two steps. First, the calculation of suitability probabilities based on the ANN were tested using the relative operating characteristic (ROC) method^71^, with an ROC value of 1 indicating that a given regression equation has the best explanatory power. Second, the kappa statistic^64,65^ was employed to evaluate the accuracy of the FLUS model by comparing the simulated and actual spatial allocation of land use in 2015. The prediction of land demand and the spatial allocation of land use during 2015–2035 under each scenario are provided in Supplementary Table S6 and Figs S4–S6.

### ES quantification

Considering the characteristics of the ecosystem, socioeconomic development, and data availability in this study area, we selected six kinds of ES, namely, water yield (WY), crop production (CP), soil conservation (SC), sand fixation (SF), carbon sequestration (CS), and aesthetic value (AV), as the research objects. The detailed variables calculation for each model and a template of the AV questionnaire are provided in Supplementary Section 2.

#### Water yield

In this study, we used the InVEST (Integrated Valuation of Ecosystem Services and Trade-offs) model to quantify and map WY^33,37,72^. The core algorithm of the InVEST model calculates the WY produced by each grid in the basin by using the principle of water balance in combination with the climate, topography, soil characteristics, and land use parameters. The calculations are as follows:

1 $${Y}_{jx}=(1-AE{T}_{xj}/{P}_{x})\times {P}_{x}$$

where *Y*_*jx*_ is the annual WY of land use type *j* in grid *x*; *AET*_*xj*_ is the average annual actual evapotranspiration of grid *x*; and *P*_*x*_ is the average annual precipitation of grid *x*.

#### Crop production

Due to inadequate habitat quality, only a very small area of oasis is suitable for agricultural development. Therefore, the cropland quantity and quality are important limiting factors for CP in Altay Prefecture. Using previous studies^73–75^ as a reference, we quantified CP by stacking the cropland quality factors onto the foundation of potential net primary productivity as follows:

2 $$CP={P}_{v}\times {I}_{zrd}$$

where *CP* represents crop production; *P*_*v*_ is the climatic productivity for a crop; and *I*_*zrd*_ is the land use level index determined by the regulations of farm land grading in China.

#### Soil conservation

In this study, SC was assessed with the Universal Soil Loss Equation (USLE), which is the most widely used model for predicting soil erosion^59,76^. SC is determined by the amount of potential soil erosion subtracted by the amount of actual soil erosion. The calculations are as follows:

3 $${A}_{c}={A}_{r}-A$$

4 $$A=R\times K\times L\times S\times C\times P$$

5 $${A}_{r}=R\times K\times L\times S$$

where *A*_*c*_ is the amount of SC; *A* is the amount of actual soil erosion; *A*_*r*_ is the amount of potential soil erosion; *R* is the rainfall erosivity factor; *K* is the soil erodibility factor; *L* is the slope length factor; *S* is the slope angle factor; and *C* and *P* represent the current vegetation cover and erosion control factors, respectively.

#### Sand fixation

We used the Revised Wind Erosion Equation (RWEQ) to evaluate the SF service in Altay Prefecture; this model takes into account the meteorological conditions, the natural conditions of underlying surfaces, and the impacts of humans on wind erosion^77–79^. The calculations are as follows:

6 $${\rm{\Delta }}Q={Q}_{0}-{Q}_{v}$$

7 $${Q}_{(x)}=(2\cdot Z/{S}^{2})\cdot {Q}_{max}\cdot {e}^{-{(x/S)}^{2}}$$

8 $${Q}_{max}=109.8\cdot (WF\times EF\times SCF\times {K}_{a}\times C)$$

9 $$S=105.71\cdot {(WF\times EF\times SCF\times {K}_{a}\times C)}^{-0.3711}$$

where Δ*Q* is the amount of SF (t · km^−2^ · a^−1^); *Q*_0_ is the amount of potential sand erosion without vegetation cover (t · km^−2^ · a^−1^); *Q*_*v*_ is the amount of actual sand erosion with vegetation cover and management (t · km^−2^ · a^−1^); *Q*_(*x*)_ is the amount of sand transported by the wind at a point *x* downwind; *Q*_*max*_ is the maximum amount of sand that can be transported downwind; and *S* is the critical field length. *WF* is the weather factor; *EF* is the soil erodibility factor; *SCF* is the soil crust factor; *K*_*a*_ is the soil roughness factor; and *C* is the vegetation cover factor.

#### Carbon sequestration

We used the InVEST model to assess CS. Taking the land use type as the evaluation unit, the carbon storage module in the InVEST model divides the ecosystem carbon stock into four basic carbon pools: aboveground biochar, underground biochar, soil carbon, and dead organic carbon. CS is calculated by multiplying the average carbon density of each of the four carbon pools with the area of each land use type^72^ as follows:

10 $${C}_{tota{l}_{j}}={C}_{abov{e}_{j}}+{C}_{belo{w}_{j}}+{C}_{soi{l}_{j}}+{C}_{dea{d}_{j}}$$

11 $$CT=\sum _{j=1}^{6}\,{C}_{tota{l}_{j}}\times {S}_{j}$$

where ${C}_{tota{l}_{j}}$ is the total carbon density and ${C}_{abov{e}_{j}}$, ${C}_{belo{w}_{j}}$, ${C}_{soi{l}_{j}}$, and ${C}_{dea{d}_{j}}$ are the aboveground carbon density, belowground carbon density, soil organic carbon density, and dead organic matter carbon density, respectively. *CT* represents the total amount of CS, and *S*_*j*_ represents the area of land use type *j*.

#### Aesthetic value

We conducted a sample questionnaire survey of residents to obtain their AV rating (integer values from 0 to 5) of different landscape types, which was taken as an indicator in evaluations of the service. First, we took photographs of different landscape types by selecting representative photographs for each landscape in summer (summer is the tourist season in Altay Prefecture, whereas spring, autumn, and winter are not suitable for traveling because of the cold weather), and these photographs were scored by respondents according to the degree of beauty. Then, the average scores of the different landscape types were calculated based on the survey results (a total of 264 valid questionnaires were surveyed), and ArcGIS was used to assign the scores to the corresponding pixels of land use types to generate layers for the AV service.

### ES changes and interactions

Based on the calculations of each ES, we used the zonal statistics tool of ArcGIS 10.2 to generate the values of each ES for the entire study area for different years under the three scenarios. In addition, we performed spatial overlay analysis of each ES between 2015 and 2035 under the different scenarios. These analyses can demonstrate future spatiotemporal changes in the ES. For the ES interactions, we first used the min-max normalization method to standardize the values of the various ES under the different scenarios for the entire study area for the period of 2015–2035. Using the ggplot2 package in R statistical software^80^, we generated flower diagrams to present trade-offs and synergies among different ES throughout future years. Many studies have used the flower diagrams to characterize the ES interactions^19,31,32^. However, the ES interactions in these studies were identified using data only for one year. In our study, we simulated the dynamic changes of ES over many years in the future and used a series of flower diagrams to characterize the multiple ES interactions. To identify the locations where ES interactions occurred, we created a set of six-digit codes and made each ES correspond to one digit. We then performed the subtraction operations on each ES in 2035 and 2015 with the ArcGIS grid calculator and used the reclassification tool to assign the value 1, 2, and 3 to increased, reduced, and unchanged pixels, respectively, for each ES. Finally, we performed spatial overlay operations on the reclassified layers and identified the multiple interactions among ES by interpreting the codes in the pixels of the output layer of overlay analysis (an example is provided in Supplementary Fig. S8). In addition, by comparing the ES interactions with the land use change layers, we further analyzed the causes for the formation of the interactions, which can provide knowledge to support ecosystem management in regions with the MODS structure.

### Data preparation

The datasets used to quantify the six ES are provided in Supplementary Table S12. In this study, ArcGIS 10.2 was used as the GIS software platform. Meteorological data are point data and need to be interpolated in ArcGIS. In Altay Prefecture, the most influential human activity on ES is land use. Soil properties and DEM changed very little over decades of years. To examine the impact of land use changes on ES, we used the multiyear average meteorological data from 2000–2015 and assumed that soil properties and the DEM will not change from 2015 to 2035. All vector and grid data were converted to the same projection coordinate system (Krasovsky_1940_Transverse_Mercator), and the grid data (except for the calculation of the SC service, for which 30 m × 30 m DEM was used) were resampled at a spatial resolution of 100 m × 100 m.

## Electronic supplementary material

Supplementary Information

## Data Availability

The datasets generated during the current study are available from the corresponding author on reasonable request.
