# Supplementary material for: Scenario analysis of ecosystem service changes and interactions in a mountain-oasis-desert system: a case study in Altay Prefecture, China
Source: Sci Rep. 2018 Aug 28;8:12939. doi: 10.1038/s41598-018-31043-y (PMC6113265; doi:10.1038/s41598-018-31043-y)
Supplement: Supplementary file 1 — Supplementary Information [file 41598_2018_31043_MOESM1_ESM.pdf]

Supplementary Information (Scientific Reports)

Scenario analysis of ecosystem service changes and interactions in a mountain-oasis-desert system: a case study in Altay Prefecture, China

Qi Fu[1,2], Ying Hou[3], Bo Wang[1,4], Xu Bi[1], Bo Li[1,2,*], and Xinshi Zhang[1,5]

[1]College of Resources Science & Technology, Faculty of Geographical Science, Beijing Normal University, Beijing, 100875, China

[2] Key Laboratory of Watershed Geographic Sciences, Nanjing Institute of Geography and Limnology, Chinese Academy of Sciences, Nanjing, 210008, China

[3]State Key Laboratory of Urban and Regional Ecology, Research Center for Eco-Environmental Sciences, Chinese Academy of Sciences, Beijing, 100085, China

[4]Institute of Ecology and Rural Environment Planning, Chinese Academy for Environmental Planning, Beijing, 100012, China

[5]Institute of Botany, Chinese Academy of Sciences, Beijing, 100093, China

[*]Corresponding author

Tel.: 86 15300200769

E-mail: libo@bnu.edu.cn

1. Land use changes simulation

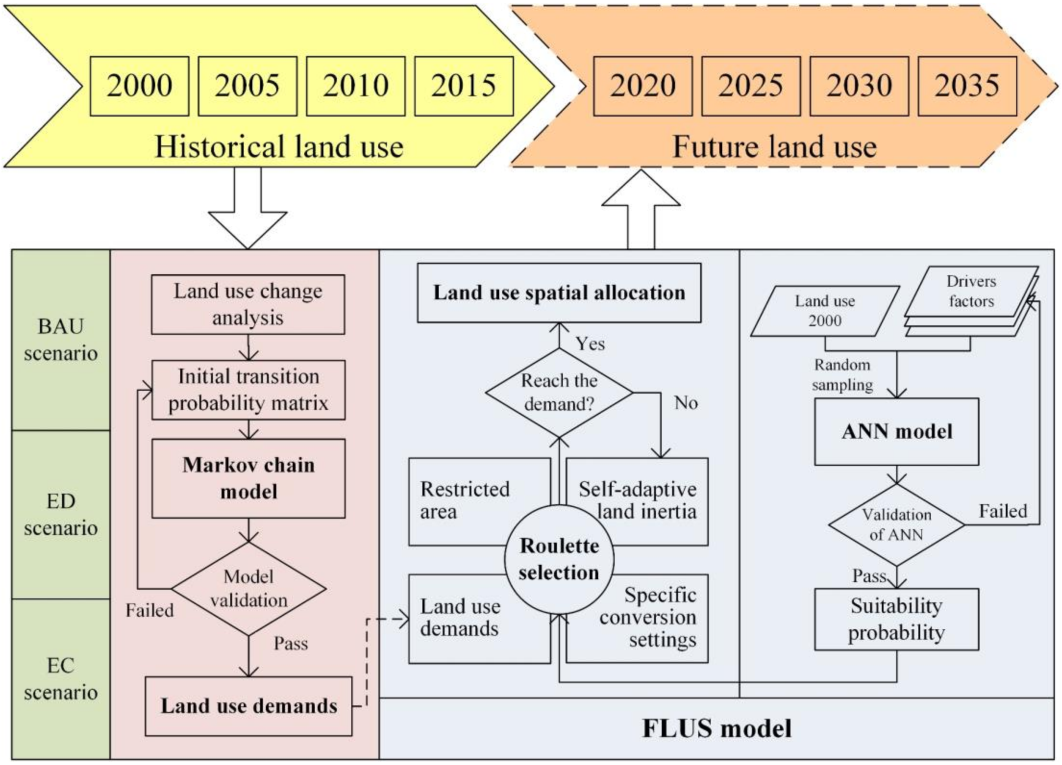

Figure S1. Flowchart of the land use change simulation.

Table S1. Factors used for training the ANN model.

Category	Factors	Data type	Year	Resolution	Data sources
Terrain	DEM	Raster	2009	30m	http://www.gscloud.cn/
	Slope	Raster	2009	30m	
	Aspect	Raster	2009	30m	
Soil	Soil depth	Raster	2000	1 km	http://westdc.westgis.ac.cn/
	PH	Raster	2000	1 km	
	Percentage of sand	Raster	2000	1 km	
	Percentage of silt	Raster	2000	1 km	
	Percentage of clay	Raster	2000	1 km	
	Soil organic carbon	Raster	2000	1 km	
	Drainage conditions	Raster	2000	1 km	
Climate	Annual mean precipitation	Point	2000–2015	–	http://cdc.cma.gov.cn/
	Annual mean temperature	Point	2000–2015	–	
Location	Distance to road	Vector	2008	–	http://midasia.data.ac.cn/
	Distance to river	Vector	2008	–	
Human influence	Population	Raster	2010	1 km	http://www.resdc.cn/
	GDP	Raster	2010	1 km	

All data were converted to raster and resampling to 100 m x 100 m spatial resolution.

Table S2. Conversion cost matrix used for simulating different land use scenarios.

		Cropland	Forest	Grassland	Water	Built-up area	Bare land
BAU scenario	Cropland	1	1	1	1	1	1
	Forest	1	1	1	1	1	1
	Grassland	1	1	1	1	1	1
	Water	1	1	1	1	1	1
	Built-up area	1	0	1	0	1	0
	Bare land	1	1	1	1	1	1
		Cropland	Forest	Grassland	Water	Built-up area	Bare land
ED scenario	Cropland	1	0	0	0	1	0
	Forest	1	1	1	1	1	0
	Grassland	1	1	1	1	1	0
	Water	1	1	1	1	1	0
	Built-up area	0	0	0	0	1	0
	Bare land	1	0	0	1	1	1
		Cropland	Forest	Grassland	Water	Built-up area	Bare land
EC scenario	Cropland	1	1	1	1	1	1
	Forest	0	1	1	1	0	0
	Grassland	0	1	1	1	0	0
	Water	0	1	1	1	0	0
	Built-up area	0	0	0	0	1	0
	Bare land	1	1	1	1	1	1

The conversion cost defines whether a conversion from one land use type (in the columns) to another (in the rows) is possible[1]. A value of 0 denotes that the conversion is not allowed, and a value of 1 denotes that the conversion is possible.

Table S3. The neighborhood weights for individual land use type under the different scenarios.

Land use types / Scenarios	Cropland	Forest	Grassland	Water	Built-up area	Bare land
BAU scenario	0.9	0.5	1	0.6	0.7	0.8
ED scenario	0.8	0.3	0.2	0.5	1	0.1
EC scenario	0.2	1	0.9	0.6	0.4	0.1

Table S4. Simulated and actual areas of land use types in 2015.

Land use types	Actual values (ha)	Simulated values (ha)	Error (%)
Cropland	422,774	421,934	-0.20%
Forest	856,034	856,038	0.00%
Grassland	4,116,909	4,117,783	0.02%
Water	198,529	198,386	-0.07%
Built-up area	22,919	22,685	-1.02%
Bare land	6,195,505	6,195,838	0.01%

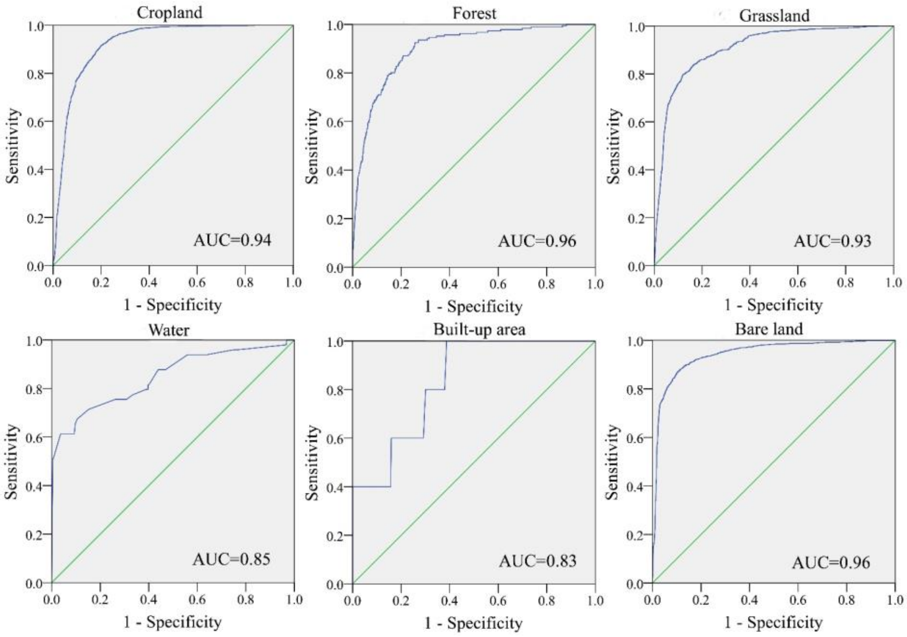

Figure S2. ROC curves and AUC values of individual land use types fitted by the ANN.

Table S5. Confusion matrix of the simulated land use pattern versus the actual pattern in 2015 (ha).

Actual / Simulated	Cropland	Forest	Grassland	Water	Built-up area	Bare land	Total
Cropland	270,877	1199	44,298	761	7838	97,801	422,774
Forest	13,746	825,409	12,853	296	953	2777	856,034
Grassland	18,617	26,429	4,033,547	1378	206	36,732	4,116,909
Water	1062	1134	7464	179,316	7	9546	198,529
Built-up area	2755	392	3902	4	11,475	4391	22,919
Bare land	115,429	1471	14,845	16,774	2440	6,044,546	6,195,505
Total	422,486	856,034	4,116,909	198,529	22,919	6,195,793	11,812,670

Kappa Coefficient = 0.94

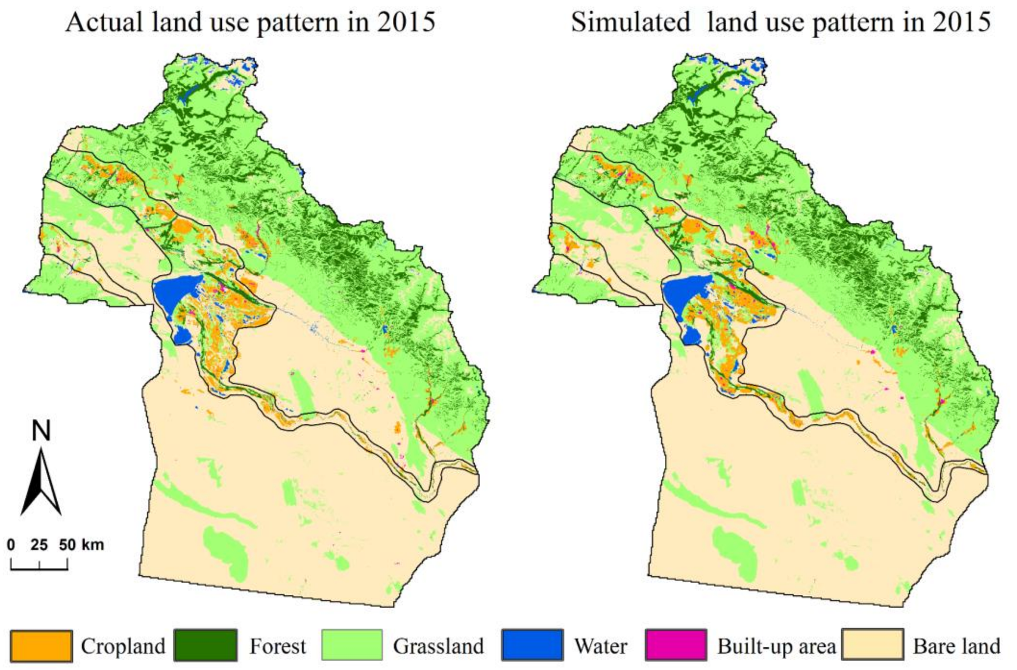

Figure S3. The simulated land use pattern and the actual land use pattern in 2015.

Table S6. The predicted land demand in the period of 2015-2035 under the different scenarios (ha)

BAU scenario	Cropland	Forest	Grassland	Water	Built-up area	Bare land
2015	422,774	856,034	4,116,909	198,529	22,919	6,195,505
2020	473,700	855,000	4,109,300	201,500	26,900	6,146,270
2025	523,400	854,000	4,102,300	204,400	31,000	6,097,570
2030	571,800	852,900	4,096,100	207,300	35,200	6,049,370
2035	619,100	851,900	4,090,600	210,100	39,500	6,001,470
ED scenario	Cropland	Forest	Grassland	Water	Built-up area	Bare land
2015	422,774	856,034	4,116,909	198,529	22,919	6,195,505
2020	500,718	854,301	4,099,805	202,089	34,559	6,121,198
2025	575,963	852,570	4,083,902	205,575	46,691	6,047,969
2030	648,588	850,844	4,069,154	208,988	59,299	5,975,797
2035	718,673	849,120	4,055,516	212,330	72,364	5,904,667
EC scenario	Cropland	Forest	Grassland	Water	Built-up area	Bare land
2015	422,774	856,034	4,116,909	198,529	22,919	6,195,505
2020	420,506	866,379	4,130,176	202,562	24,720	6,168,327
2025	418,270	876,656	4,143,370	206,545	26,513	6,141,316
2030	416,067	886,867	4,156,492	210,479	28,296	6,114,469
2035	413,895	897,013	4,169,542	214,364	30,070	6,087,786

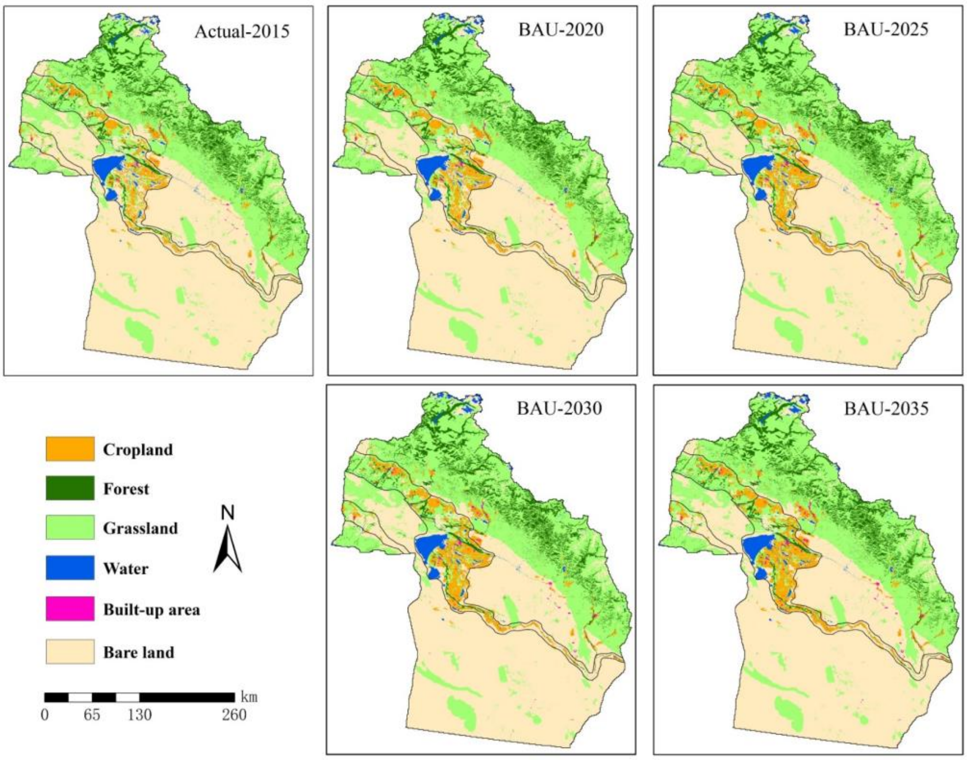

Figure S4. The simulated land use pattern under BAU scenario from 2015 to 2035.

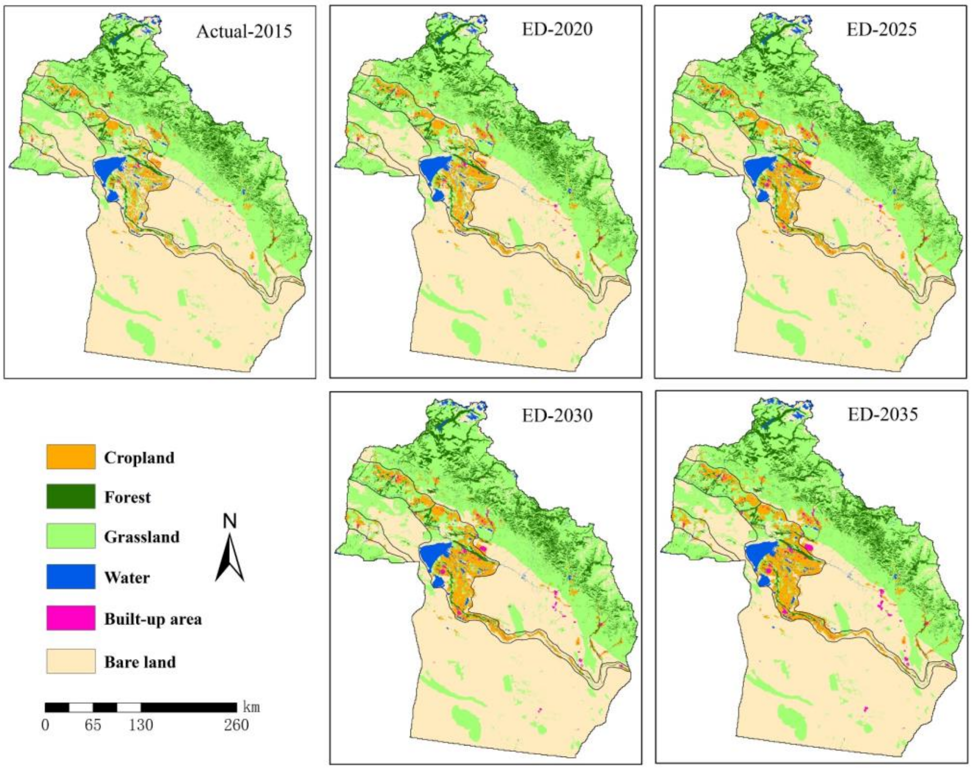

Figure S5. The simulated land use pattern under ED scenario from 2015 to 2035.

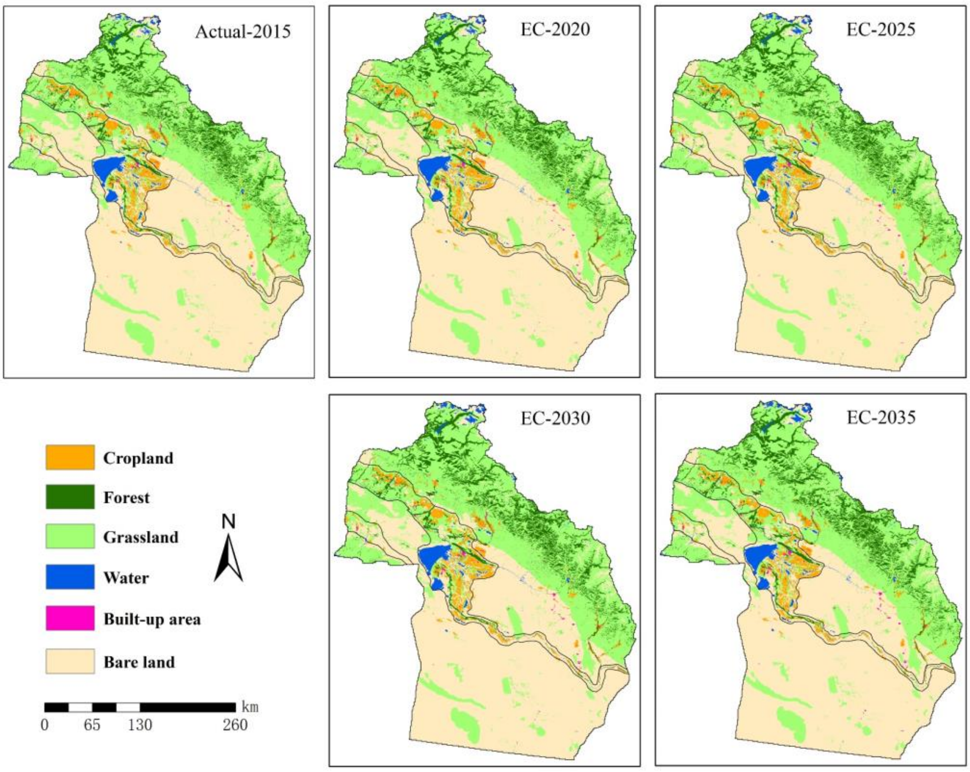

Figure S6. The simulated land use pattern under EC scenario from 2015 to 2035.

2. ES quantification

2.1 Water yield

Table S7. The variables calculation for water yield in the InVEST model

Variables	Equations	Description
AET_{xj}/P_x	$\frac{AET_{xj}}{P_x} = \frac{(1+\omega_x R_{xj})}{(1+\omega_x R_{xj} + 1/R_{xj})}$	AET_{xj}/P_x approximates the Budyko curve developed by Zhang *et al.*[2]; R_{xj} is the Budyko aridity index (dimensionless) of grid *x*; ω_x is a dimensionless ratio characterizing the natural climate and soil properties.
R_{xj}	$R_{xj} = K_{xj} \times \frac{ET_0}{P_x}$	P_x is the average annual precipitation (mm) of grid *x* and is generated by interpolation[3] base on ArcGIS 10.2. ET_0 is the potential evapotranspiration (mm). K_{xj} is the coefficient of vegetation evapotranspiration and is derived from the InVEST user's guide[4].
ET_0	$ET_0 = 0.0013 \times 0.408 \times RA \times (T_{avg} + 17) \times (TD - 0.0123P_m)^{0.76}$	ET_0 is calculated based on the 'modified Hargreaves' equation. RA is extraterrestrial radiation (MJ·m^{2}·d^{-1}); T_{avg} is the average of the mean daily maximum and mean daily minimum temperatures (°C); TD is the difference between mean daily maximum and mean daily minimum temperatures (°C); P_m is the average monthly precipitation (mm).

ω_x	$\omega_x = Z \times \frac{AWC_x}{P_x}$	Z is the Zhang coefficient[2], which ranges from 1 to 10 and needs to be calibrated with monitoring data from the local hydrometric stations. In our study, when the Zhang coefficient is equal to 9, the deviation for simulated and observed water yield is the smallest (7.8%). AWC_x is the volumetric (mm) plant available water content.
AWC	$AWC = Min(Soil\ \ Depth, Root\ \ Depth) \times PAWC$	The soil depth data came from the Second National Soil Survey of China. The root depth data refer to the study of Canadell et al.[5]
$PAWC$	$PAWC = 54.509 - 0.132SAN\% - 0.003(SAN\%)^2 - 0.055SIL\% - 0.006(SIL\%)^2 - 0.738CLA\% + 0.007(CLA\%)^2 - 2.688OM\% + 0.501(OM\%)^2$	$SAN\%$, $CLA\%$, $SIL\%$, and $OM\%$ represent the measured contents of sand, clay, silt and organic matter (%), respectively.

2.2 Crop production

Table S8. The variables calculation for crop production

Variables	Equations	Description
P_v	$P_v = RDI^2 \times \frac{r \times (1+RDI+RDI^2)}{(1+RDI)\times(1+RDI^2)} \times Exp(-\sqrt{9.87 + 6.25RDI})$	This equation was proposed by Zhou and Zhang[6], which is more suitable for arid regions when compared with the models of Miami, Thornthwaite, etc. RDI is the radiation dryness.
RDI	$RDI = (0.629 + 0.237 \times \frac{58.93BT}{P} - 0.00313 \times (\frac{58.93BT}{P})^2)^2$	BT is the mean annual biological temperature (°C); P is the average annual precipitation (mm).
BT	$BT = \frac{\sum t}{365}\ \ or\ \ BT = \frac{\sum T}{12}$	t represents the average daily temperature which is greater than 0 °C and less than 30 °C; T represents the average monthly temperature which is greater than 0 °C and less than 30 °C.
I_{zrd}	$I_{zrd} = \frac{\sum_{i=1}^{m} w_i \times f_i}{100}$	w_i is the weight of factor i; f_i is the index value of factor i. They were evaluated based on the Regulation for Gradation on Agriculture Land Quality in China[7].

2.3 Soil conservation

Table S9. The variables calculation for soil conservation in the USLE model

Variables	Equations	Description
R	$R = \sum_{i=1}^{12}(1.735 \times 10^{1.5\times\frac{{P_m}^2}{P} - 0.8188})$	This equation was derived from Wischmeier and Smith[8], where P is the average annual precipitation (mm); P_m is the average monthly precipitation (mm).
K	$K = 0.01383 + 0.51575K_{epic}$ $K_{epic} = \left\{0.2 + 0.3exp\left[-0.0256SAN(1 - \frac{SIL}{100})\right]\right\} \times (\frac{SIL}{CLA+SIL})^{0.3} \times \left[1 - \frac{0.25OM}{OM+\exp(3.72-2.95OM)}\right] \times \left[1 - \frac{0.7SN}{SN+\exp(-5.51+22.9SN)}\right]$	The soil erodibility factor K was determined using the erosion-productivity impact calculator (EPIC) model[9] and was corrected according to the study of Zhang *et al*[10]. SAN, CLA, SIL, and OM represent the measured contents of sand, clay, silt and organic matter (%), respectively. $SN = 1 - SAN/100$

L	$L = (\frac{\lambda}{22.13})^m$	λ represents the slope length and can be calculated through the Hydrology and Distance modules of ArcGIS9.3. m represents slope length index.
m	$m = \begin{cases} 0.2, & slope \le 1\% \\ 0.3, & 1\% < slope \le 3.5\% \\ 0.4, & 3.5\% < slope \le 5\% \\ 0.5, & 5\% < slope \le 9\% \\ \frac{\beta}{1+\beta}, & slope \ge 9\% \\ \beta = \frac{\sin\theta}{0.0986\times(3\sin\theta^{0.8}+0.56)} \end{cases}$	$slope$ is the percentage slope.
S	$S = \begin{cases} 10.8\sin\alpha + 0.03, & \alpha < 5° \\ 16.8\sin\alpha - 0.5, & 5° < \alpha \le 10° \\ 21.9\sin\alpha - 0.96, & \alpha > 10° \end{cases}$	α represents the slope.
C and P	Refer to the previous studies[11–13].	In this study, the C values for cropland, forest, grassland, water, built-up area, and bare land are 0.3, 0.006, 0.04, 0, 0, 1, respectively; the P values for cropland, forest, grassland, water, built-up area, and bare land are 0.3, 1, 1, 0, 0, 1.

2.4 Sand fixation

Table S10. The variables calculation for sand fixation in the RWEQ model

Variables	Equations	Description
WF	$WF = \frac{\sum_{i=1}^{N} WS_i(WS_i - WS_t)^2 \times N_d \times \rho}{N \times g} \times SW \times SD$	This equation was derived from Fryrear et al.[14] WS_i is wind speed at 2 meters (m/s); WS_t is threshold wind speed at 2 meters (assumed 5 m/s); N is the number of wind speed observations (normally 500); N_d is the number of days in time period; ρ is air density (kg/m^3); g is acceleration due to gravity (m/s^2); SW is the soil wetness, dimensionless; SD is the snow cover factor, dimensionless.
EF	$EF = \frac{29.09+0.31SAN+0.17SIL+\frac{0.33SAN}{CLA}-2.59OM}{100}$	SAN, CLA, SIL, and OM represent the measured contents of sand, clay, silt and organic matter (%), respectively.
SCF	$SCF = \frac{1}{1 + 0.0066CLA^2 + 0.21OM^2}$	CLA and OM represent the measured contents of clay and organic matter (%), respectively.
C	$C = e^{a_i \times SC}$	SC is annual average vegetation coverage (%).a_i is the coefficient of different vegetation types; the values of cropland, forest, grassland, water, built-up area, and bare land are -0.0438, -0.1535, -0.1151, 0, 0, and -0.0768, respectively.
K_a	$K_a = e^{(1.86K_r - 2.41K_r^{0.934} - 0.127C_r)}$<br>$K_r = 0.2 \times \frac{(\Delta H)^2}{L}$	K_r is the roughness of soil ridge; C_r is the random roughness factor; L is the ground fluctuation parameter; ΔH is the altitude difference within the range of L, which could be calculated based on Neighborhood Statistics tools in ArcGIS.

The equations of EF, SCF, C, and K_a refer to Ouyang et al.[15]

2.5 Carbon sequestration

Table S11. Carbon density of each land use type in Altay Prefecture.

Land use types	Carbon density (t/ha)		
	C_{above}	C_{below}	C_{soil}
Cropland	0.57	8.07	10.84
Forest	4.24	11.59	23.69
Grassland	3.53	8.65	9.99
Water	0	0	0
Built-up area	1.2	0	7.1
Bare land	0	0	7.8

The carbon density refer to the InVEST user's guide[4] and the study by Chen *et al*. [16]

2.6 Aesthetic value

Figure S8 shows a template of the aesthetic value questionnaire used in our study. We took photographs of the different landscape types and selected representative photographs for each landscape. These photographs were scored by 264 respondents according to the degree of beauty (integer values from 0 to 5). A total of 20 landscape types were used for scoring in the questionnaire. These landscape types were classified as 6 land use types: cropland, forest (including dense forest, shrub, sparse woodland, and tree nursery), grassland (including alpine meadow, mountain grassland, and desert grassland), water body (including river, lake, reservoir, and glacier), built-up area (including urban land, rural settlement, and factory), and bare land (including sandy land, Gobi Desert, saline-alkali land, barren earth, and bare exposed rock). Based on the scored photographs, we calculated the mean scores of the 20 landscape types and then used the area weighting method to calculate the AV scores of the 6 land use types. The main intent of this study is to obtain and map the local residents' intuitive perceptions of the landscape aesthetics. In addition, because we had limited time for the survey and the majority of the residents in the study area are Kazakh and speak very little Mandarin, we did not record specific indicators related to the interviewees' own attributes (e.g., age, gender, work and income). In spite of this, the survey investigated the local residents' perceptions of landscape aesthetics, which met the needs of this study.

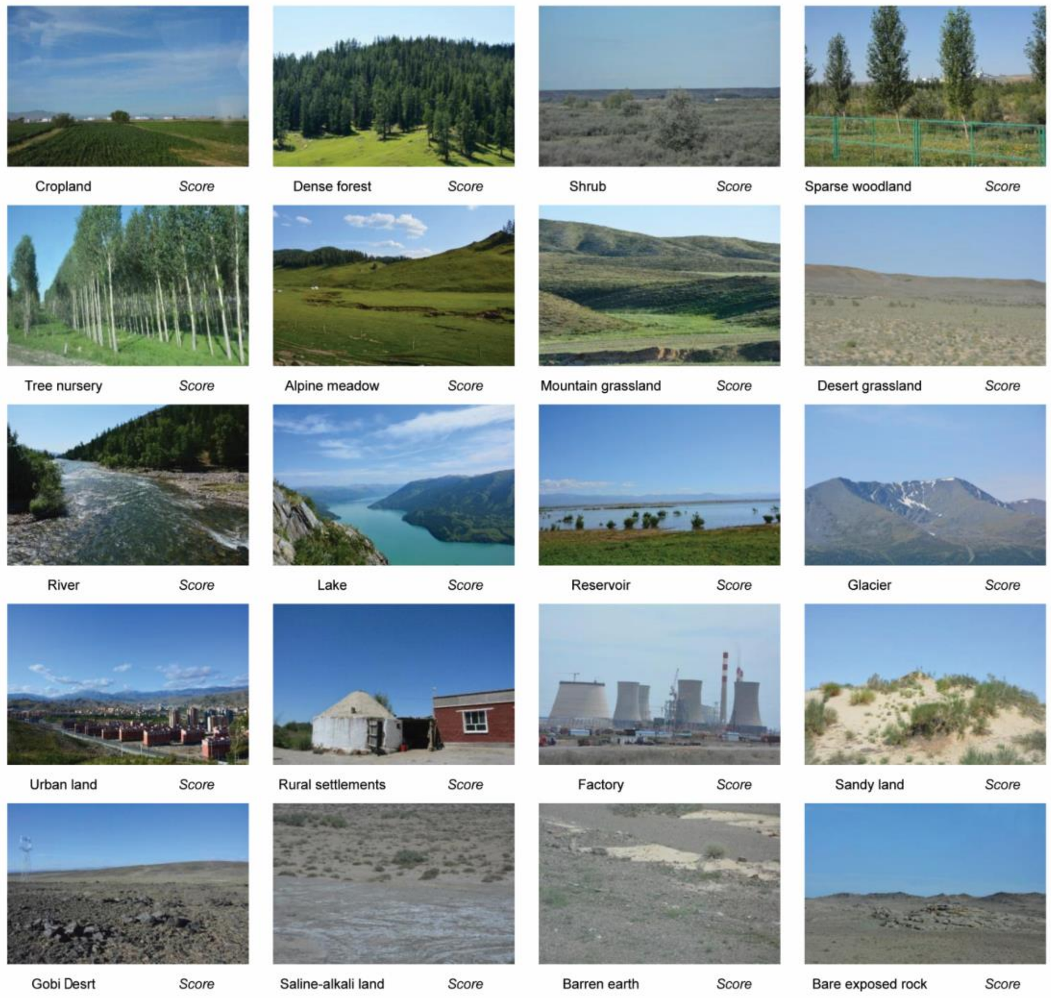

Figure S7. A template of the aesthetic value questionnaire used in Altay Prefecture.

Table S12. Datasets used for calculating ES.

Data	Data type	Spatial resolution	Time scale	Description	Data source
Meteorological data	Point	–	2000–2015	The meteorological data include annual precipitation, annual temperature, monthly precipitation, monthly temperature, daily temperature, daily radiation, and daily wind speed. These data were used for calculating WY, CP, SC, and SF.	http://cdc.cma.gov.cn/
Soil data	Raster	1 km	2000	Soil data contains many attributes, including surface soil texture, organic matter content, PH, thickness of soil layer, irrigation conditions, degree of salinity, and drainage conditions, which were used for calculating the WY, CP, SC, and SF.	http://westdc.westgis.ac.cn/
Land use/ land cover maps	Raster	100 m	2000, 2005, 2010, 2015	Land use/ land cover maps were used for calculating all the six ES.	http://www.resdc.cn/
DEM	Raster	30 m	2009	DEM was used for calculating the CP, SC, and SF. Moreover, the slope and aspect layers were generated according to DEM.	http://www.gscloud.cn/
MODIS NDVI	Raster	250 m	2000–2015	The NDVI data was used for calculating the SF service.	http://www.gscloud.cn/
Hydrological monitoring data	Table	–	1990–2015	This data was used to calibrate Z value when calculating the WY service.	Water Conservancy Bureau of Altay Prefecture
Carbon density	Table	–	2005	Carbon density was used for calculating the CS service.	Refer to InVEST user's guide[4] and the study of Chen *et al.* [16]
Scores of different landscape types.	Table	–	2015	This data was used for calculating the AV service.	Random questionnaire survey

Basic geographic information data	Vector	–	2008	The basic geographic information data include boundaries, water systems and roads in Altay Prefecture.	http://midasia.data.ac.cn/

WY, CP, SC, SF, CS, and AV refer to water yield, crop production, soil conservation, sand fixation, carbon sequestration, and aesthetic value, respectively.

3. ES interactions

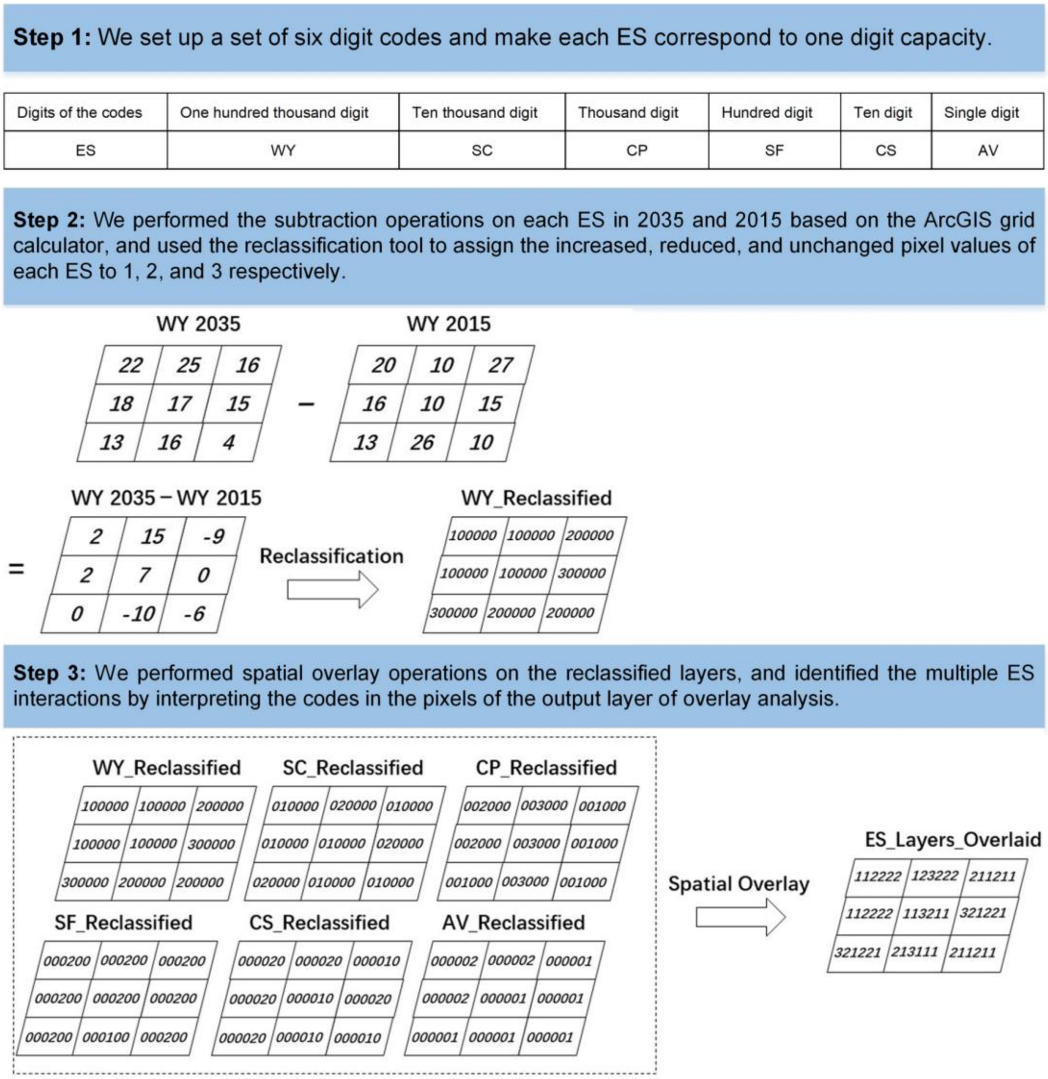

Figure S8. An example for identifying the locations where multiple interactions among ES occurred: the code "112222" indicates that WY and SC increased simultaneously (synergies), CP, SF, CS, and AV decreased simultaneously (synergies), and the two services WY and SC both exhibited trade-off interactions with the four services CP, SF, CS, and AV.

Table S13. The area and causes for the formation of the multiple interactions among ES.

Scenarios	Codes	Multiple ES interactions	Area (km^2)	The area proportion of ES interactions in different MODS zones			Causes
				M_zone	O_zone	D_zone	
BAU	211211	SC+ CP+ CS+ AV+, WY- SF-	1911	8%	91%	1%	The conversion of bare land to cropland.
	213111	SC+ SF+ CS+ AV+, WY-	355	77%	17%	6%	The conversion of grassland to forest in the M_zone and O_zone, and the conversion of bare land to grassland in the D_zone.
	123222	WY+, SC- SF- CS- AV-	350	51%	19%	30%	The conversion of forest to grassland in the M_zone and O_zone, and the conversion of grassland to bare land in the D_zone.
	121222	WY+ CP+, SC- SF- CS- AV-	128	27%	73%	0%	The conversion of forest to cropland.
	112222	WY+ SC+, CP- SF- CS- AV-	90	46%	54%	0%	The conversion of cropland to built-up area.
	221221	CP+ AV+, WY- SC- SF- CS-	82	26%	74%	0%	The conversion of grassland to cropland.
	122122	WY+ SF+, SC- CP- CS- AV-	48	38%	48%	15%	The conversion of cropland to bare land.
ED	211211	SC+ CP+ CS+ AV+, WY- SF-	2517	10%	87%	3%	The conversion of bare land to cropland.
	213111	SC+ SF+ CS+ AV+, WY-	960	90%	10%	0%	The conversion of grassland to forest in the M_zone and O_zone, and the conversion of

							bare land to grassland in the D_zone.
	123222	WY+, SC- SF- CS- AV-	917	91%	8%	0%	The conversion of forest to grassland in the M_zone and O_zone, and the conversion of grassland to bare land in the D_zone.
	221221	CP+ AV+, WY- SC- SF- CS-	528	22%	78%	0%	The conversion of grassland to cropland.
	113211	WY+ SC+ CS+ AV+, SF-	211	55%	36%	9%	The conversion of bare land to built-up area.
	112222	WY+ SC+, CP- SF- CS- AV-	185	50%	50%	0%	The conversion of cropland to built-up area.
	121222	WY+ CP+, SC- SF- CS- AV-	105	25%	75%	0%	The conversion of forest to cropland.
	233321	AV+, WY- CS-	84	85%	13%	2%	The conversion of bare land to water.
EC	213111	SC+ SF+ CS+ AV+, WY-	1623	68%	22%	10%	The conversion of grassland to forest in the M_zone and O_zone, and the conversion of bare land to grassland in the D_zone.
	123222	WY+, SC- SF- CS- AV-	438	93%	6%	0%	The conversion of forest to grassland in the M_zone and O_zone.
	233321	AV+, WY- CS-	110	83%	11%	6%	The conversion of bare land to water.
	112112	WY+ SC+ SF+ CS+, CP- AV-	87	48%	51%	1%	The conversion of cropland to grassland.
	211211	SC+ CP+ CS+ AV+, WY- SF-	66	8%	92%	0%	The conversion of bare land to cropland.
	113211	WY+ SC+ CS+ AV+, SF-	56	61%	34%	5%	The conversion of bare land to built-up area.
	122122	WY+ SF+, SC- CP- CS- AV-	48	17%	77%	6%	The conversion of cropland to bare land.

Because the area of Altay Prefecture is approximately 118,000 km^2, only those multiple interactions occurring in pixels (100 m × 100 m) with a summed area of over 40 km^2 are shown in this table. M_zone, O_zone, and D_zone represent the mountain zone, oasis zone, and desert zone, respectively.

References

1. Liu, X. *et al.* A future land use simulation model (FLUS) for simulating multiple land use scenarios by coupling human and natural effects. *Landsc. Urban Plan.* **168,** 94–116 (2017).
2. Zhang, L., Dawes, W. R. & Walker, G. R. Response of mean annual evapotranspiration to vegetation changes at catchment scale. *Water Resour. Res.* **37,** 701–708 (2001).
3. Jing, W., Feng, M. & Yang, Y. A statistical downscaling approach of NCEP/NCAR reanalysis temperature data. *J. Geo-information Sci.* **15,** 819–828 (2013).
4. Sharp, R. *et al. InVEST User Guide*. *The Natural Capital Project, Stanford University, University of Minnesota, The Nature Conservancy, and World Wildlife Fund.* (2016). doi:10.1007/s13398-014-0173-7.2
5. Canadell, J. *et al.* Maximum rooting depth of vegetation types at the global scale. *Oecologia* **108,** 583–595 (1996).
6. Zhou, G. & Zhang, X. Study on NPP of natural vegetation in China under global climate change. *Acta Phytoecol. Sin.* **20,** 11–19 (1996).
7. China National Standardization Management Committee. in (2012).
8. Wischmeier, W. H. & Smith, D. D. *Predicting rainfall erosion losses from crop land east of the rocky mountains*. (Washington, DC: USDA-ARS, 1965).
9. Sharpley, A. N. & Williams, J. R. EPIC-erosion/productivity impact calculator: 1. Model documentation. *Tech. Bull. - United States Dep. Agric.* **4,** 206–207 (1990).
10. Zhang, K., Peng, W. & Yang, H. Soil erodibility and its estimation for agricultural soil in China. *Acta Pedol. Sin.* **44,** 7–13 (2007).
11. Liu, A., Wang, J. & Liu, Z. Remote sensing quantitative monitoring of soil erosion in Three Gorges Reservoir area: a GIS/RUSLE based research. *J. Nat. Disasters* **18,** 25–30 (2009).
12. Yu, F., Li, X., Chen, Y., Wang, H. & Yang, M. Land use change and soil erosion evaluation in Huangfuchuan Watershed. *Acta Ecol. Sin.* **26,** 1947–1956 (2006).
13. Cai, C., Ding, S., Shi, Z., Huang, L. & Zhang, G. Study of applying USLE and geographical information system IDRISI to predict soil erosion in small watershed. *J. Soil Water Conserv.* **14,** 19–24 (2000).
14. Fryrear, D. W. *et al.* RWEQ: Improved wind erosion technology. *J. Soil Water Conserv.* **55,** 183–189 (2000).
15. Ouyang, Z. *et al.* Improvements in ecosystem services from investments in natural capital. *Science (80-.).* **352,** 1455–1459 (2016).
16. Chen, Y. *et al.* Sources and sinks of carbon from forest land use change in Xinjiang, China during 1975-2005. *Geogr. Res.* **32,** 1987–1999 (2013).
